# Decision-making of Jordanian dentists when restoring root canal-treated molars: a cross-sectional vignette survey

**DOI:** 10.3389/fdmed.2026.1903315

**Published:** 2026-08-10

**Authors:** Sanaa Aljamani, Rasha A. Alamoush, Aseel M. Sharaireh, Mohammad AL-Rabab’ah, Shanil Rajesh Patel, Emad Moawad, Fadi Jarad

**Affiliations:** 1 Department of Restorative Dentistry, School of Dentistry, The University of Jordan, Amman, Jordan; 2 Department of Fixed and Removable Prosthodontics, School of Dentistry, The University of Jordan, Amman, Jordan; 3 Department of Endodontics, School of Dentistry, University of Liverpool, Liverpool, United Kingdom

**Keywords:** clinical decision-making, endodontics, indirect restoration, molars, restorative dentistry, root canal treatment

## Abstract

**Aims:**

This study aimed to assess the decision-making of Jordanian dentists when restoring root canal-treated molars (RCTMs) and identify the tooth-related factors influencing restorative treatment decisions.

**Methodology:**

A previously validated vignette survey, adapted from a UK study, was distributed electronically to registered dentists in Jordan between August and December 2024. The survey comprised three clinical scenarios: UR7 (one remaining wall), LL7 (three remaining walls), and LL6 (one remaining wall with a crack), representing different degrees of structural tooth compromise. Associations between dentist characteristics and restorative preferences were analysed using Fisher–Freeman–Halton exact tests,

**Results:**

A total of 364 responses were analysed. Participants comprised 50.8% general dental practitioners and 49.2% specialists; 63% had ≤10 years of experience. Crowns were the preferred restoration for the more structurally compromised molars (Scenarios 1 and 3), whereas onlays predominated in Scenario 2. Zirconia was the most frequently selected material among respondents choosing indirect restorations. Remaining walls, ferrule, marginal ridge loss, and tooth position were the most influential factors, whereas skeletal/lateral guidance and periodontal status were less frequently considered. No statistically significant differences were observed between dentist groups in restorative choices or material preferences (*P* > 0.05).

**Conclusion:**

Jordanian dentists demonstrated broadly consistent decision-making, with indirect restorations generally preferred across the three clinical scenarios. However, variation in consideration of some tooth-related factors suggests opportunities to better align clinical decision-making with evidence-based recommendations.

## 1 Introduction

Restoring endodontically treated molars (RCTMs) remains a clinical challenge due to the substantial structural compromise these teeth often exhibit. Molars, being key load-bearing teeth without successors, are particularly vulnerable to caries and fractures (1, 2). Loss of marginal ridges and cusps, combined with the removal of dentin during root canal therapy, significantly reduces tooth stiffness and increases the risk of biomechanical failure (3–5). While root canal treatment addresses pulpal and periradicular pathology, long-term tooth survival is largely influenced by the quality of the coronal restoration (6). Studies indicate that treatment failure often arises from factors such as cracks and caries, emphasising the critical role of coronal restoration (3, 7–10).

A wide range of restorative options is available for RCTMs, including direct resin composites, indirect onlays, and full coverage crowns. Advances in restorative materials such as monolithic zirconia and lithium disilicate have expanded treatment possibilities; however, uncertaintyremains regarding the most appropriate restorative approach for different clinical situations because long-term evidence remains heterogeneous (11–14). Studies often differ in design, methodology, and follow-up duration, making direct comparisons difficult. Consequently, clinicians must frequently rely on their clinical judgment when selecting a restorative approach, particularly in the absence of universally accepted evidence-based guidelines, as highlighted by the European Society of Endodontology position statement (15).

The long-term success of restoring root canal-treated molars depends not only on the type of restorative approach but also on comprehensive assessment of tooth-related factors such as the number of remaining walls, loss of marginal ridges, ferrule effect, occlusion, periodontal status, and tooth position (16–22). The European Society of Endodontology emphasizes that restorative treatment planning should preserve tooth structure, maintain occlusal function, minimize microleakage, and support periodontal health while considering biomechanical and aesthetic principles (15). Similarly, Bhuva et al. emphasized that restorations must preserve occlusal function, minimize microleakage, and maintain periodontal health while being guided by biomechanical and aesthetic principles (23). However, little is known about **how clinicians prioritize these factors when making restorative decisions in standardized clinical scenarios**, particularly among dentists particularly particularly within the Jordanian dental community.

Previous surveys conducted in Jordan have described dentists' restorative preferences for root canal-treated molars; however, they have not investigated how clinicians integrate multiple tooth-related factors into restorative decision-making using standardized clinical scenarios (24). Consequently, important questions remain regarding the relative importance clinicians assign to structural and biological factors when planning the restoration of RCTMs.

The present study aimed to evaluate Jordanian dentists' clinical decision-making regarding RCTM using standardized vignette scenarios and to identify the tooth-related factors that most strongly influenced restorative treatment planning. We hypothesized that restorative decision-making and the prioritization of tooth-related factors would vary according to dentists' qualifications and clinical experience.

## 2 Methodology

### 2.1 Study design and ethical approval

This cross-sectional electronic vignette survey was conducted among registered dentists practicing in Jordan to evaluate clinical decision-making regarding the restoration of root canal-treated molars. Ethical approval for this study was obtained from the University of Jordan Ethics Committee (Decision No. 80-2022). The study was reported in accordance with the Checklist for Reporting Results of Internet E-Surveys (CHERRIES).

### 2.2 Survey instrument and clinical scenarios

A previously validated vignette survey, originally developed and administrated using the JISC online survey platform (Bristol, UK) (25), was adapted to suit the Jordanian clinical context. The adaptation was limited to minor modifications in wording and terminology to ensure cultural and professional relevance, while preserving the original clinical scenarios, accompanying radiographs and intraoral scans, questionnaire structure, and study objectives. The adapted questionnaire was pilot tested with 20 practicing dentists to assess its clarity and relevance prior to dissemination. Responses obtained during the pilot phase were excluded from the original data analysis.

The survey included three clinical scenarios representing common endodontically treated molar cases. Each scenario provided detailed information on tooth-related factors, such as the number of remaining walls, loss of marginal ridges, presence of a ferrule, periodontal status, and tooth position, supported by post-endodontic radiographs and intraoral scans. Where applicable, additional clinical information relating to occlusal or parafunctional considerations was also incorporated into the scenarios to reflect realistic treatment planning.

Participants were instructed to to base their restorative decisions solely on thesetooth-related factors, assuming successful root canal treatment and excluding patient-related factors like cost or patient preference. Occlusal and parafunctional habits were provided only when relevant to the specific clinical scenario.

Participants selected their preferred restorative option for each clinical scenario using multiple-choice questions with nominal categorical response options (see Table 1). For questions exploring the tooth-related factors influencing their decision-making, respondents were permitted to select more than one applicable option where appropriate.

**Table 1:** Represents the characteristics of each scenario: intra-oral scanner images captured after the removal of existing restoration and caries, but before the completion of RCT.

Clinical scenario	Summary of the clinical scenario	Factors involved
Scenario 1 (UR7)	Medically fit 57-year-old male with a compromised UR7 with chronic/asymptomatic apical periodontitis. The tooth was non-mobile, with no obvious cracks or localised pocketing. The patient had a class 2 division 1 occlusion and good oral hygiene, was a bruxist and did not wear a mouthguard. 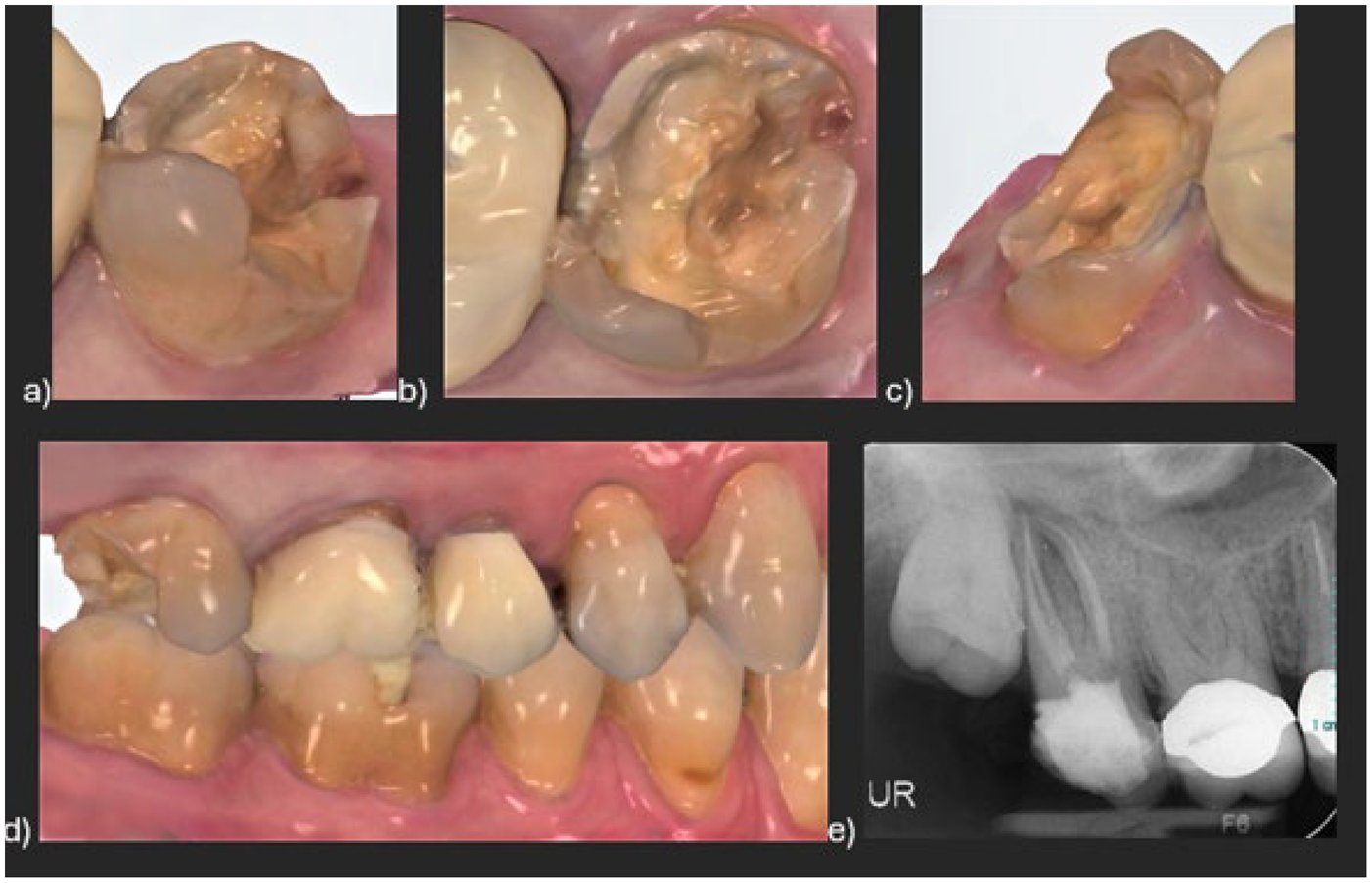	Remaining number of walls Thickness of remaining walls Parafunction Loss of proximal contacts Tooth position
Scenario 2 (LL7)	48-year-old female with well-controlled asthma presenting with chronic/asymptomatic apical periodontitis LL7. There were no clinical signs of mobility, localised pocketing or cracks, and the patient was periodontally stable. She had a class 2 division 1 occlusion and no parafunctional habits. 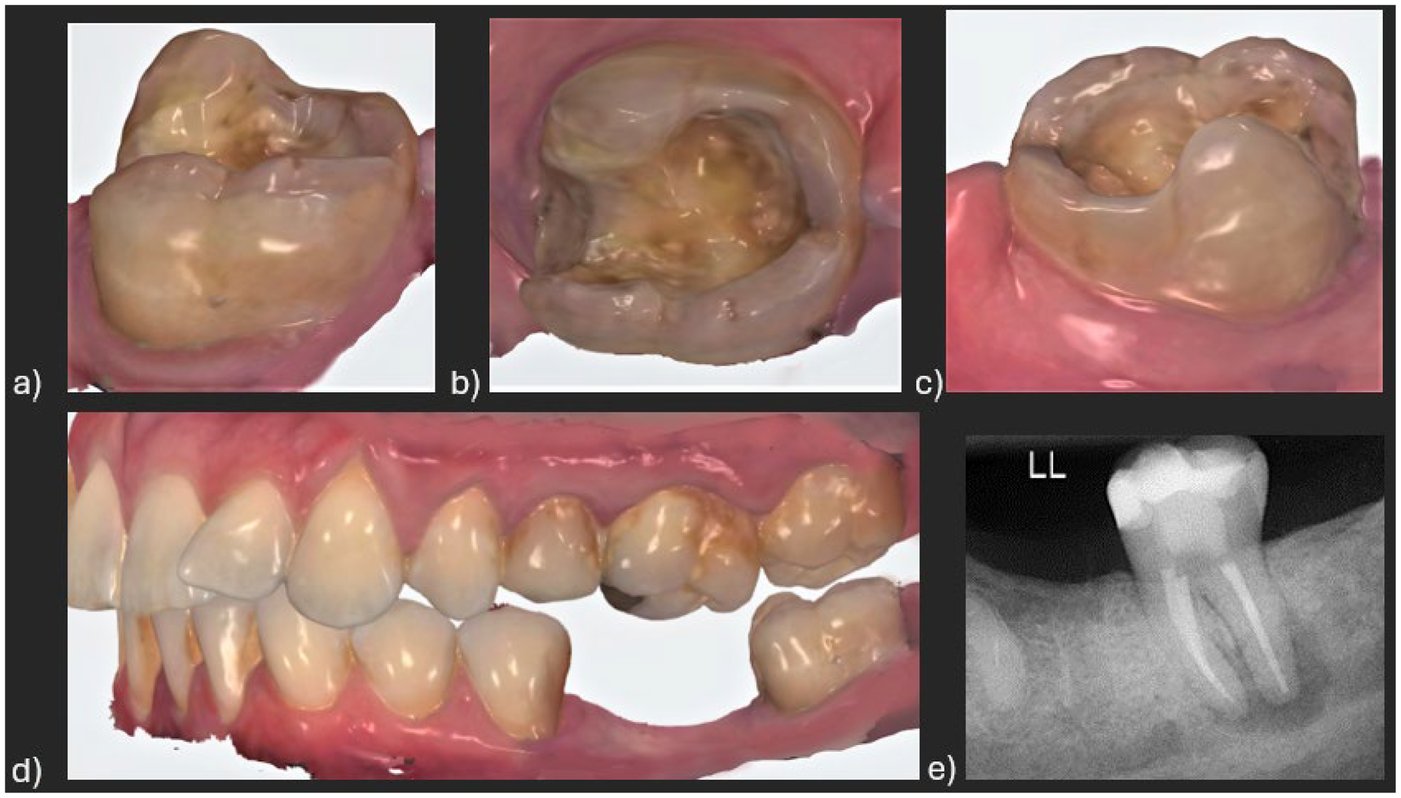	Remaining number of walls Thickness of remaining walls Loss of proximal contacts Tooth position
Scenario 3 (LL6)	57-year-old female suffering with hay fever, asthma and epilepsy had chronic/asymptomatic apical periodontitis LL6. Clinically there was no mobility or localised pocketing, but a crack was present running down the mesial margin however did not extend to the pulp chamber floor. The patient had localised plaque-induced periodontitis with BPE scores 311/120, a class 1 occlusion and did not suffer from bruxism 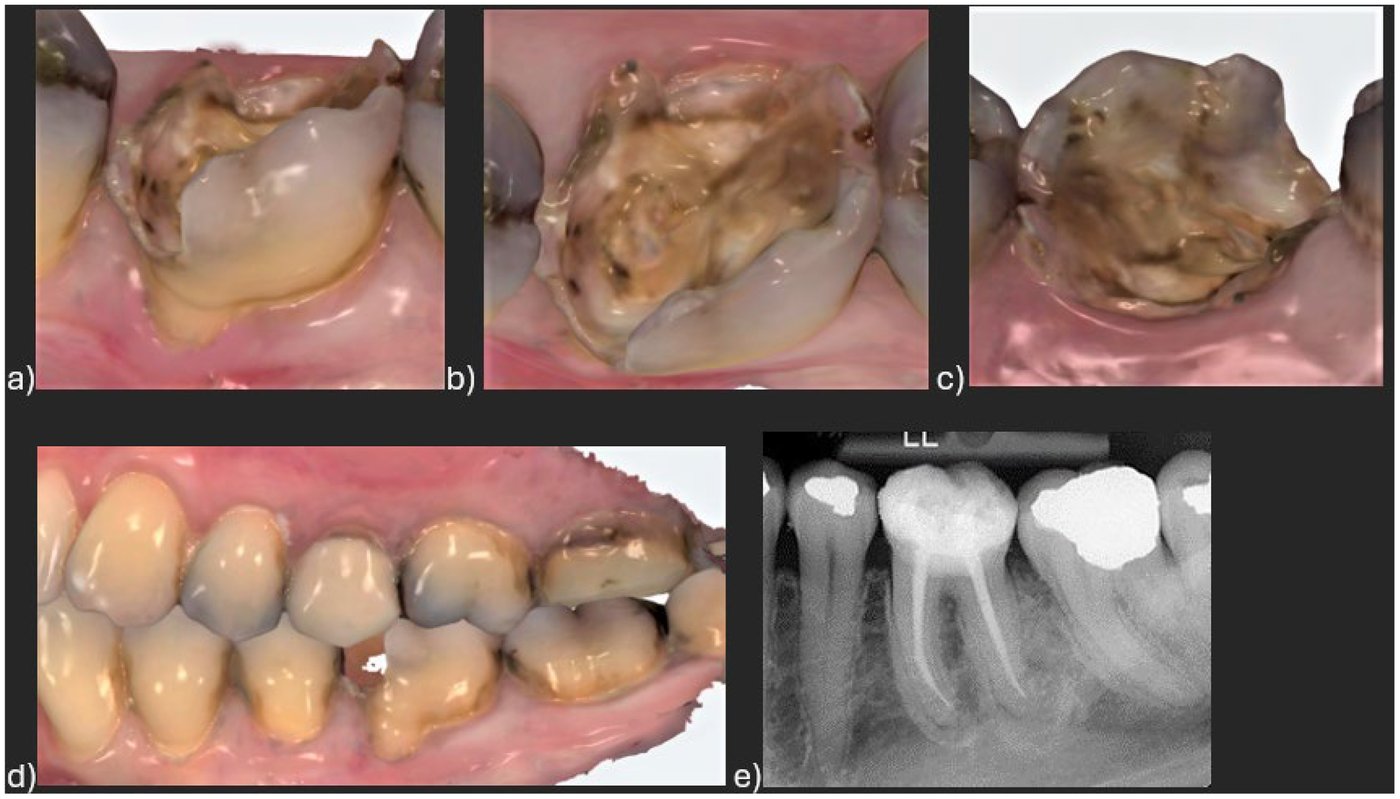	Remaining number of walls Thickness of remaining walls Cracks Periodontal health

For each case scenario: (a) buccal; (b) occlusal; (c) palatal/lingual; (d) bite view; and (e) periapical radiograph following endodontic completion. The coronal cavities remained unchanged in size for all cases during root canal treatment of each case (25).

### 2.3 Survry distribution and data analysis

The survey was distributed online via social media platforms (WhatsApp, Facebook) and email to registered Jordanian dentists between August to November 2024. Participation was voluntary and anonymous, and completion of the questionnaire implied informed consent. Measures were implemented to minimize duplicate submissions by restricting one response per device/IP address through the JISC survey platform. Only fully completed questionnaires were recorded by the platform and included in the final analysis.

The minimum required sample size was calculated using Raosoft® sample size online tool (Raosoft Inc., Seattle, WA, USA), assuming a population of approximately 6.500 registered dentists, a 95% confidencelevel, a 5% margin of error, yeiding a minimum sample size of 363 responses.

Data were analysed using IBM SPSS Statistics version 27 (IBM Corp., Armonk, NY, USA). Descriptive statistics were used to summarize participant demographics and response frequencies. Associations between dentist groups (qualification, years of experience, and speciality) and treatment choices were evaluated using the Fisher–Freeman–Halton exact test with Monte Carlo simulation, as several contingency tables contained small, expected cell counts. Pearson's chi-square statistics were examined during the preliminary analysis to assess the suitability of exact testing; however, the Fisher–Freeman–Halton exact test was adopted as the primary inferential method where chi-square assumptions were not met. Statistical significance was set at *P* < 0.05.

## 3 Results

**Figure 1:**
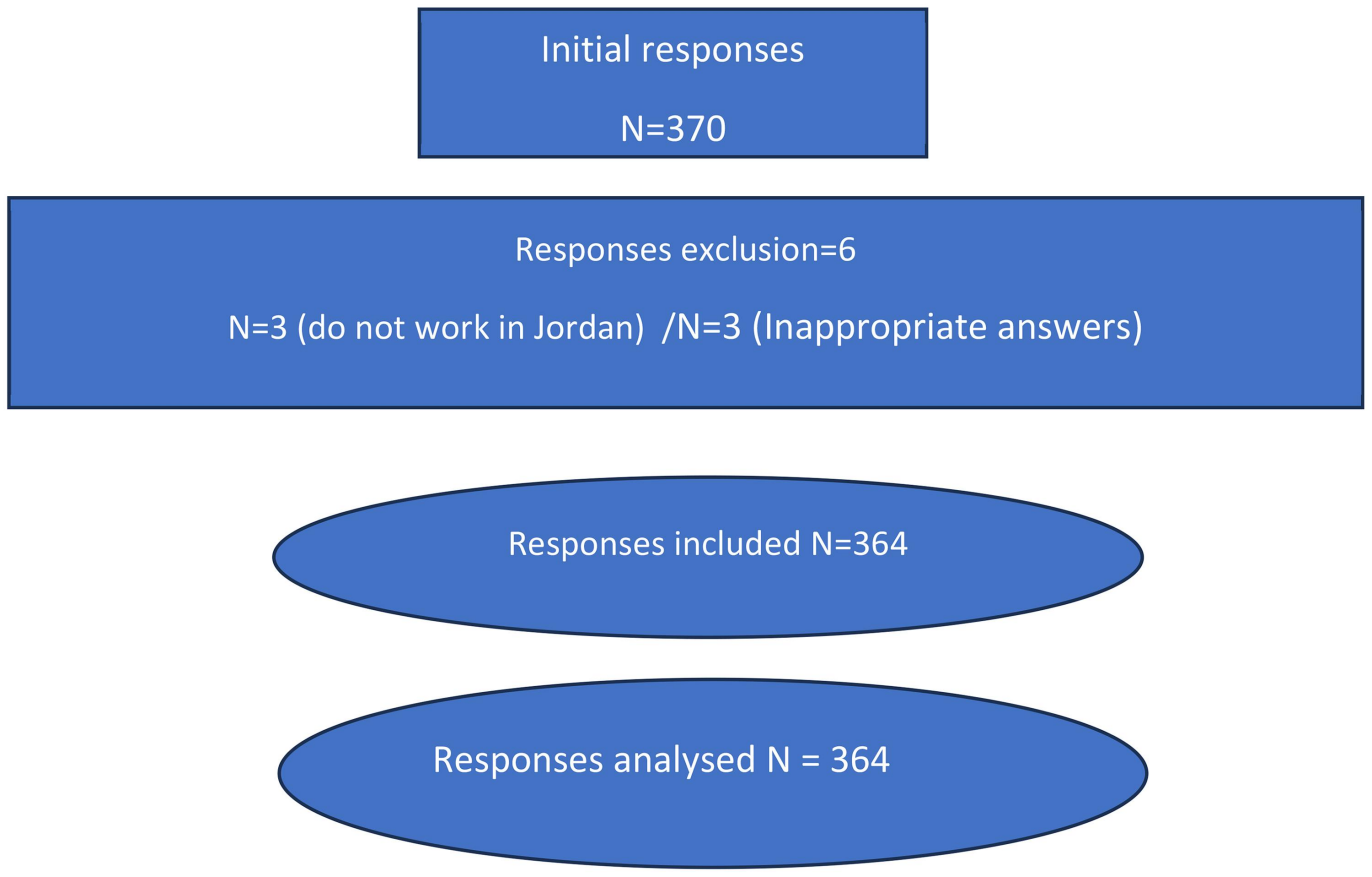
Flowchart illustrates participants inclusion and exclusion.

A total of 370 questionnaire responses were received during the study period. The survey was distributed electronically through multiple open channels,the number of dentists who viewed or received the survey invitation could not be determined; therefore, a traditional response rate could not be reliably calculated. Following data screeningcollection, six responses were excluded due to not meeting predefined eligibility criteria. Thus, 364 responses were analysed. The participant selection process is summarized in Figure 1.

### 3.1 Demographic data and participant grouping

**Figure 2:**
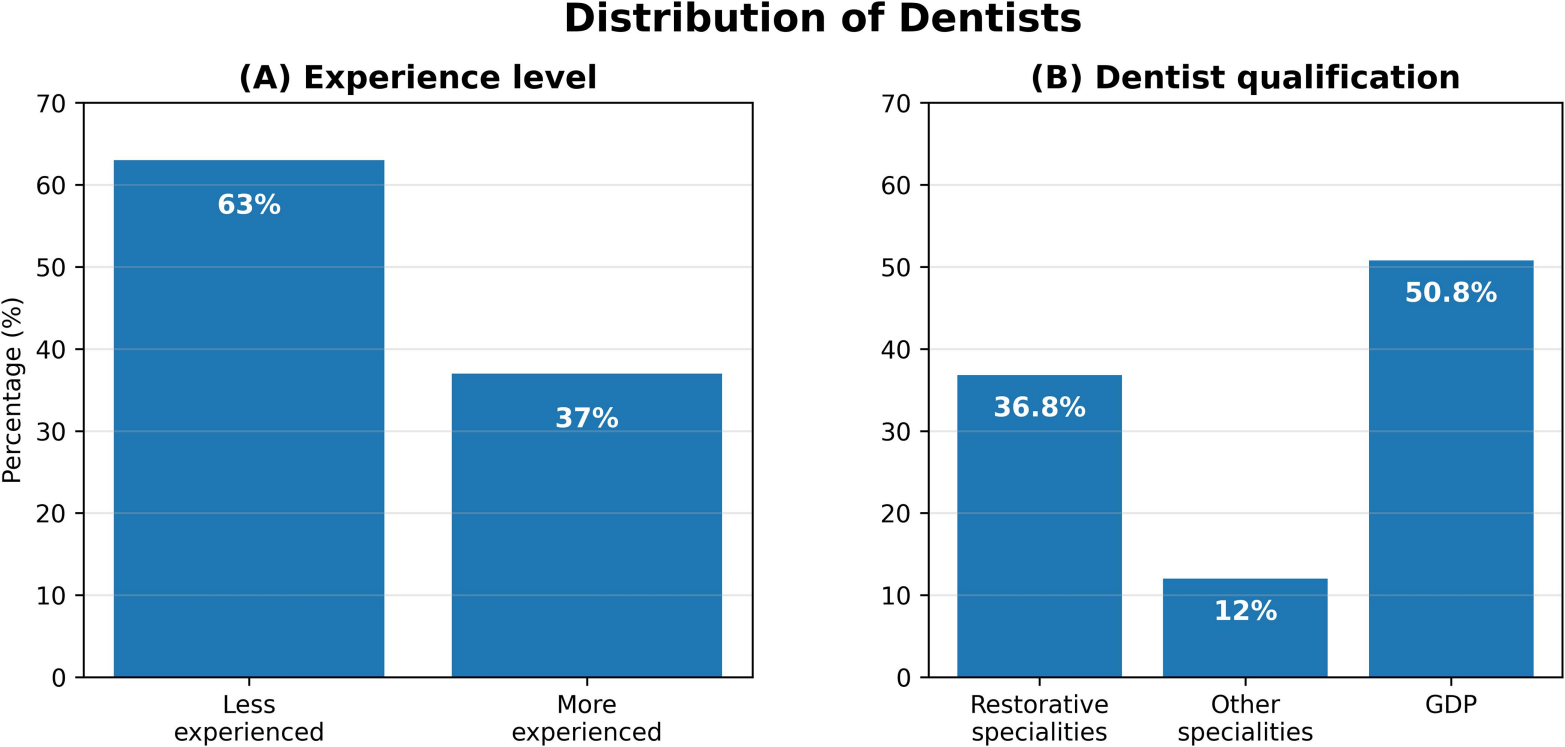
A bar chart showing the distribution of dentists in this survey by 2 categories; **(A)** experience level -highlighted in orange and **(B)** speciality level-highlighted in blue. the percentage in each category sums up to 100% which is the full percentage of the study sample.

Participants were grouped by qualification (General Dental Practitioners vs. Specialists), experience (0–10 years vs. >10 years), and speciality relevance to restoration of RCTMs (Restorative vs. Non-restorative).Regarding dentist background knowledge, data of the restorative relevant speciality (i.e., Endodontists, Prosthodontists, Restorative dentists, Aesthetic dentists) were separated from the data of the non-restorative relevant speciality (Oral surgery, Oral medicine, Periodontists, Paediatric dentists, and special care dentistry.Participant demographic characteristics are presented in Figure 2.

Most dentists (58%) practised dentistry in the private sector, followed by university practice (20%), with the remaining dentists working in the armed forces, Ministry of Health, and mixed practice settings. Table 2 shows the use of magnification for assessing tooth restorability in each category and work environment.

**Table 2:** Detailed table shows the sample characteristics; the use of magnification in each category and the working environment of the dentists participated in the study.

Sample characteristics	Number of dentists (%)
Use of magnification	
less experience	45 (19.70%)
More experience	55 (40.40%)
Restorative specialities	75 (55%)
Other specialities	0 (0%)
GDP	25 (13.50%)
Working environment	
Private sector	58% (211)
University setting	20% (72)
Armed forces, Health services	12% (43)
Mixed private and public services	10% (36)

### 3.2 Clinical scenarios analysis

For descriptive purposes, restorative options were grouped into direct restorations, full-coverage crowns, conservative indirect restorations (onlays and endocrowns), and extraction where selected. These groupings were used solely to facilitate presentation of the survey findings and do not imply a hierarchy of clinical appropriateness.

#### 3.2.1 Scenario 1: restoration of UR7

**Q1) For the question “How would you restore UR7”?** and **Q2) “If you chose an Indirect restoration, what material would you use to restore UR7?”**

For Scenario 1 (UR7), crowns were the most frequently selected restorative option across both experience groups. However, a statistically significant association was observed between years of clinical experience and restoration choice (Fisher–Freeman–Halton exact test, *P* = 0.044), indicating differences in restorative preferences according to experience. Among participants choosing an indirect restoration, zirconia was the most preferred restorative material, followed by metal-ceramic restorations. No statistically significant associations were identified between restoration choice or restorative material and dentists' qualification, years of clinical experience, or speciality (see Tables 3, 4)

**Table 3:** The decision made regarding the type of restoration for UR7 by different categories of dentists.

Dentist categories	Direct restoration	Crown	Onlay	Endo crown	Extraction	Total	*P*-value
A) Less experienced	14 (6.1%)	110 (48.3%)	28 (12.3%)	73 (31.9%)	3 (1.2%)	228	P^*^ = 0.044
More experienced	11 (8.1%)	52 (38.2%)	21 (15.4%)	47 (34.6%)	5 (3.7%)	136	
B) Restorative specialities	5 (3.7%)	53 (39.6%)	17 (12.7%)	55 (41.1%)	4 (3.0%)	134	*P* = 0.198
Other specialities	4 (8.9%)	24 (53.3%)	10 (22.2%)	7 (15.6%)	0 (0.0%)	45	
GDP	16 (8.7%)	85 (45.9%)	22 (11.9%)	58 (31.4%)	4 (2.2%)	185	

The percentages in Parentheses represent the percent of selected restoration types from each category *P*-values obtained using the Fisher–Freeman–Halton exact test with Monte Carlo simulation.

^*^Indicates a statistically significant difference (*P* < 0.05).

**Table 4:** The decision made regarding the restoration material selected for the indirect restoration of UR7.

Dentist categories	Indirect composite	Gold	Hybrid ceramic	Lithium disilicate	Metal	Metal-ceramic	Zirconia	*P*-value
A) Less experienced	2 (0.9%)	20 (8.8%)	8 (3.5%)	40 (17.4%)	11 (4.8%)	56 (24.6%)	74 (32.5%)	*P* = 0.07
More experienced	1 (0.7%)	13 (9.6%)	6 (4.4%)	24 (17.6%)	7 (5.1%)	26 (19.1%)	43 (31.6%)	
B) Restorative specialties	2 (1.5%)	11 (8.2%)	6 (4.5%)	28 (20.9%)	3 (2.2%)	28 (20.9%)	47 (35.1%)	*P* = 0.213
Other specialties	0 (0.0%)	3 (6.7%)	0 (0.0%)	7 (15.6%)	6 (13.3%)	10 (22.2%)	15 (33.3%)	
GDP	1 (0.5%)	19 (10.3%)	8 (4.3%)	8 (16.2%)	5 (2.7%)	49 (26.5%)	75 (40.5%)	

The percentages in Parentheses represents represent the percent of selected material from each category. *P*-values obtained using the Fisher–Freeman–Halton exact test with Monte Carlo simulation.

#### 3.2.2 Scenario 2: restoration of LL7

**Q1) For the question “How would you restore LL7”**? and **Q2)** **“If you chose an Indirect restoration, what material would you use to restore LL7?”**

For Scenario 2 (LL7), onlays were the most frequently selected restorative option across all participant groups. Among respondents selecting an indirect restoration, zirconia was the most preferred restorative material, followed by metal-ceramic restorations, consistent with the findings observed in Scenario 1. No statistically significant associations were identified between restoration choice or restorative material and dentists' qualification, years of clinical experience, or speciality (see Tables 5, 6).

**Table 5:** The decision made regarding the type of restoration for LL7 by different categories of dentists.

Dentist categories	Direct restoration	Crown	Onlay	Endo crown	Extraction	Total	*P* value
A) Less experienced	56 (24.6%)	47 (20.6%)	72 (31.6%)	47 (20.6%)	6 (2.6%)	228	*P* = 0.638
More experienced	32 (23.5%)	33 (24.3%)	43 (31.6%)	25 (18.4%)	3 (2.2%)	136	
B) Restorative specialities	32 (23.9%)	29 (21.6%)	51 (38.1%)	19 (14.2%)	3 (2.2%)	134	*P* = 0.171
Other specialities	9 (20.0%)	8 (17.8%)	15 (33.3%)	11 (24.4%)	2 (4.4%)	45	
GDP	47 (25.4%)	43 (23.2%)	49 (26.5%)	42 (22.7%)	4 (2.2%)	185	

The percentages in Parentheses represent the percent of selected restoration types from each category. *P*-values obtained using the Fisher–Freeman–Halton exact test with Monte Carlo simulation.

**Table 6:** The decision made regarding the restoration material selected for the indirect restoration of LL7.

Dentist categories	Indirect composite	Gold	Hybrid ceramic	Lithium disilicate	Metal	Metal-ceramic	Zirconia	*P*-Value
A) Less experienced	3 (1.3%)	4 (1.7%)	9 (3.9%)	50 (21.9%)	1 (0.5%)	20 (8.8%)	79 (34.7%)	*P* = 0.075
More experienced	2 (1.5%)	3 (2.2%)	4 (2.9%)	27 (21.2%)	2 (1.5%)	17 (11.8%)	48 (31.1%)	
B) Restorative specialties	2 (1.5%)	4 (2.9%)	3 (2.2%)	31 (26.1%)	3 (2.2%)	10 (6.7%)	47 (39.6%)	*P *= 0.16
Other specialties	2 (4.4%)	0 (0.0%)	3 (6.7%)	8 (17.8%)	1 (2.2%)	8 (17.8%)	12 (26.7%)	
GDP	1 (0.5%)	3 (1.6%)	7 (3.8%)	38 (20.5%)	1 (0.5%)	19 (10.3%)	68 (36.7%)	

The percentages in Parentheses represents the percent of selected material from each category. *P*-values obtained using the Fisher–Freeman–Halton exact test with Monte Carlo simulation.

#### 3.2.3 Scenario 3: restoration of LL6

**Q1) For the question “How would you restore LL6”? and Q2) “If you chose an Indirect restoration, what material would you use to restore LL6?”**

or Scenario 3 (LL6), crowns represented the most frequently selected restorative option across all participant groups, although tooth extraction was also commonly selected, particularly among dentists with fewer years of clinical experience and those from non-restorative specialties. Among respondents selecting an indirect restoration, zirconia was the most frequently preferred restorative material, followed by metal-ceramic restorations. No statistically significant associations were identified between restoration choice or restorative material and dentists' qualification, years of clinical experience, or speciality (see Tables 7, 8).

**Table 7:** The decision made regarding the type of restoration by different categories of dentists.

Dentist category	Direct restoration	Crown	Onlay	Endo crown	Extraction	Total	*P*-value
A) Less experienced	6 (2.6%)	100 (43.9%)	12 (5.3%)	33 (14.5%)	77 (33.8%)	228	*P* = 0.213
More experienced	7 (5.2%)	53 (39.0%)	12 (8.8%)	21 (15.4%)	43 (31.6%)	136	
B) Restorative specialities	7 (5.3%)	69 (52.3%)	9 (6.8%)	19 (14.2%)	34 (22.7%)	134	*P* = 0.161
Other specialities	2 (4.4%)	15 (33.3%)	1 (2.2%)	8 (17.8%)	19 (42.2%)	45	
GDP	4 (2.2%)	74 (40.0%)	14 (7.6%)	27 (14.6%)	66 (35.7%)	185	

The percentages in parentheses represent the percent of selected restoration types from each category. *P*-values obtained using the Fisher–Freeman–Halton exact test with Monte Carlo simulation.

**Table 8:** The decision made regarding the restoration material selected for the indirect restoration of LL6.

Dentist categories	Composite indirect composite	Gold	Hybrid ceramic	Lithium disilicate	Metal	Metal ceramic	Zirconia	*P*-value
A) Less experienced	2 (0.9%)	7 (3.1%)	6 (2.6%)	22 (9.6%)	7 (3.1%)	41 (26.3%)	60 (34.6%)	*P* = 0.213
More experienced	1 (0.7%)	6 (4.4%)	4 (2.9%)	14 (10.3%)	3 (2.2%)	19 (13.9%)	41 (30.1%)	
B) Restorative specialities	0 (0.0%)	4 (3.1%)	4 (3.1%)	15 (11.4%)	4 (3.1%)	22 (16.7%)	43 (32.6%)	*P* = 0.313
Other specialties	0 (0.0%)	1 (2.2%)	1 (2.2%)	2 (4.4%)	2 (4.4%)	5 (11.1%)	13 (28.9%)	
GDP	1 (0.5%)	8 (4.3%)	5 (2.7%)	19 (10.3%)	4 (2.2%)	33 (17.8%)	45 (24.3%)	

The percentages in Parentheses represents the percent of selected material from each category. *P*-values obtained using the Fisher–Freeman–Halton exact test with Monte Carlo simulation.

### 3.3 Tooth related factors selection in each clinical scenario

**Figure 3:**
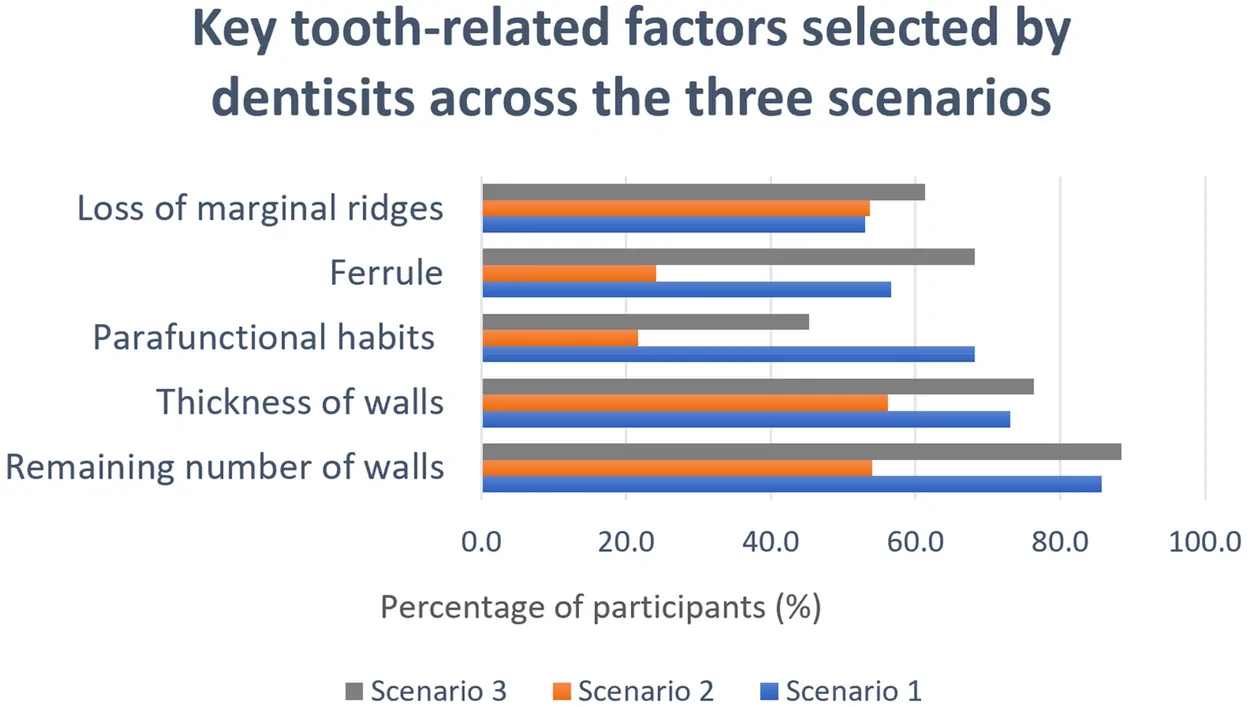
Percentage of participants selecting key tooth-related factors considered during restorative decision-making for root canal-treated molars across the three clinical scenarios (UR7, LL7, and LL6).

**Figure 4:**
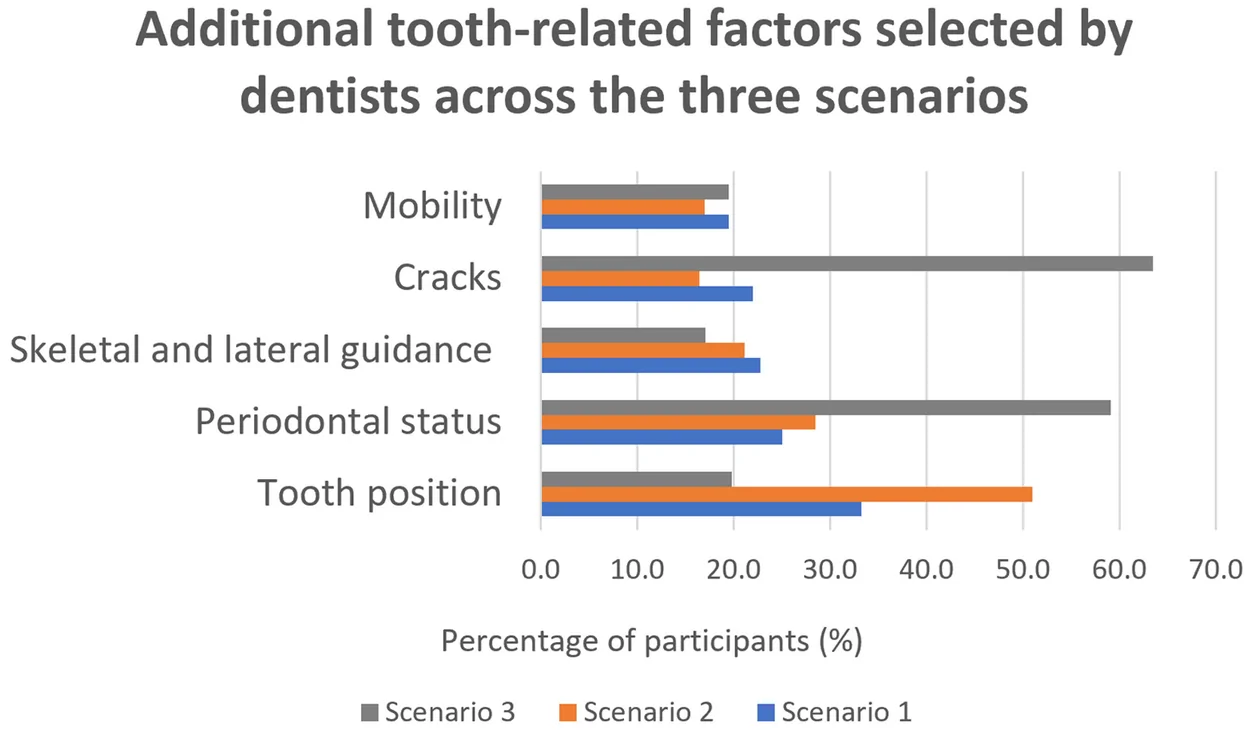
Percentage of participants selecting additional tooth-related factors considered during restorative decision-making for root canal-treated molars across the three clinical scenarios (UR7, LL7, and LL6).

Figures 3, 4 summarize the tooth-related factors considered by participants when selecting a restorative approach across the three clinical scenarios. As shown in Figure 3, the most frequently selected key factors were the number of remaining walls and the thickness of the remaining tooth structure, particularly in Scenario 3. Ferrule presence and loss of marginal ridges were also commonly identified as important determinants of treatment planning. Parafunctional habits were selected more frequently in Scenario 1 than in the other scenarios.

Among the additional factors (Figure 4), tooth position and periodontal status were the most considered variables across all scenarios. Cracks were selected more frequently in Scenario 3, whereas mobility and skeletal guidance were the least frequently reported factors overall.

## 4 Discussion

This present study evaluated the restorative decision-making for endodontically treated molars among Jordanian dentists using an online validated vignette questionnaire that is previously utilised in the UK (25). Overall, the findings of this study demonstrated a considerable level of agreement in restorative treatment planning across the three clinical scenarios, with indirect restorations preferred over direct restorations. Crowns were the predominant choice for the more structurally compromised teeth (Scenarios 1 and 3), whereas onlays were preferred when greater coronal tooth structure remained (Scenario 2). These findings closely mirror those reported in the original UK study (25), suggesting that restorative decision-making is primarily driven by tooth-related structural factors rather than geographical or healthcare system differences.

Across the three clinical scenarios, restorative decisions were largely consistent across dentisits with different qualifications and specialties. However, a significant association between years of clinical experience and restorative choice was observed in Scenario 1, suggesting that clinical experience may influence decision-making in more structurally compromised cases. Nevertheless, treatment planning across the three scenarios was primarily driven by tooth-related structural factors, particularly the number and thickness of the remaining walls. This is consistent with previous reports and the ESE position statement that emphasises on preservation of sound tooth structure and appropriate cuspal coverage restoration following RCT (18, 20, 22, 26).

Direct restorations were selected more frequently in Scenario 2, likely reflecting the greater amount of remaining tooth structure. Although direct restorations are not typically recommended as definitive options for RCTMs, they are often used as core build-up materials (28). The preference for onlays in this scenario suggests more conservative approach. Evidence comparing long-term outcomes of different restorative approaches remains scarce due to variation in study designs, follow-up periods, and patient populations (27).

Zirconia was the preferred restorative materialfor most dentists in all three scenarios. This contrasts the UK vignette study, in which gold and lithium disilicate were more freqenly selected in the same scenarios (25). The predominance of zirconia in the present study may reflect contemporary restorative practice, increasing aesthetic demands, and material availability. Although bilayered zirconia restorations remain susceptible to veneering fracture, particularly in patients with parafunctional habits, monolithic zirconia has demonstrated favourable mechanical performance and acceptable wear characteristics (28–30).

Lithium disilicate was the second most selected restorative material of choice for dentists in all categories in scenario two. This could be justified by the amount of tooth structure left that favours adhesive restoration for adequate bonding to the remaining tooth structure (26). In contrast, metal-ceramic restorationswere more commonly selectedin the third scenario, wherelimited remaining tooth and a guardedprognosis may have opted for conventional full coverage restoration. Most dentists did not consider gold for indirect restoration despite its superior fracture resistance, probably reflecting regional differences in esthetic expectations, material availability and clinical practice patterns when compared to the UK study (25, 31).

Although the present study hypothesized that dentists' clinical experience and speciality would influence restorative decision-making, this hypothesis was only partially supported. While a significant association between years of clinical experience and restorative choice was observed in Scenario 1, restorative decisions were otherwise largely consistent across the different clinician groups. Overall, these findings suggest that treatment planning for endodontically treated molars is driven primarily by tooth-related structural characteristics rather than clinician demographics.

The tooth related factors evaluated in this study were based on ESE guidelines recommendations for RCTMs (15). In this study, these factors were categorised into key factors (e.g., structural integrity indicators like remaining walls and ferrule) and additional factors (e.g., parafunction, periodontal condition, and occlusal guidance) to better understand their relative influence on clinical decision-making. Participants consistently prioritised the number and thickness of the remaining walls across all three scenarios, highlighting the importance of preserving coronal tooth structure when planning definitive restorations.This aligns with existing literature highlighting the significance of preserving the remaining tooth structure for the clinical success of endodontically treated teeth (32).

However, periodontal status, ferrule, tooth position, occlusal guidance, and parafunctional habits appeared to receive less attention despite their recognised influence on restorative prognosis. Periodontal health is particularly important where ferrule is limited or restoration margins extend subgingivally, and the absence of periodontal attachment loss has been associated with improved treatment outcomes (33). These findings suggest that although clinicians appropriately prioritised structural integrity, a more comprehensive assessment of biological and functional factors may further optimise restorative decision-making for RCTMs.The findings of this vignette survey are consistent with those of a previous survey that targeted a similar group of dentists in Jordan (24). The main difference in this study is the presentation of three distinct scenario scenarios with specific factors designed to assess whether these factors influence treatment decisions, as opposed to a general, non-case-based survey (24). Compared to the UK study (25), Jordanian dentists favoured indirect restorations as the primary treatment option for all three scenarios. Importantly, both studies identified the preservation of remaining tooth structure as the principal determinant of restorative decision-making, despite differences in clinician populations and healthcare settings. However, Jordanian dentists demonstrated a greater preference for zirconia, whereas the UK cohort selected gold and lithium disilicate more frequently in selected scenarios, suggesting that material selection may be influenced by regional practice patterns,patient expectations and material availability (25). These comparable results in both populations underscore the growing recognition of the benefits in restoring RCTMs, but further research and evidence-based guidelines are essential to help clinicians make informed decisions regarding material selection and treatment planning for RCTMs.

This study has several limitations. First, the survey was distributed electronically through multiple professional and social media platforms which intitles a conviencience sampling; therefore, a conventional response rate could not be calculated, like the original UK vignette study (25). Also, this recruitment strategy may have introduced a selection bias. Second, the cross-sectional findings were based on self-reported responses to standardized clinical scenarios and may not fully reflect real-world clinical decision-making. Although the vignette design allowed standardized assessment of restorative choices, it could not incorporate all patient-related factors encountered in clinical practice. Finally, despite achieving the required sample size, some subgroup analyses involved relatively small numbers of participants and should therefore be interpreted with caution.

Future research should focus on clinical outcome studies comparing different restoration types, along with qualitative investigations to understand the rationale behind dentists' decisions across various professional categories. To support more advanced data analysis and improve generalizability larger and more representative samples would improve the generalisability of the findings and enable more robust subgroup analyses across different healthcare settings.

## 5 Conclusion

This vignette survey demonstrated that restorative decision-making for root canal-treated molars among Jordanian dentists was guided primarily by tooth-related structural characteristics rather than clinician experience or speciality, although clinical experience influenced restorative choice in one clinical scenario. Indirect restorations were generally preferred, with treatment decisions largely reflecting the amount of remaining tooth structure and the complexity of each clinical scenario.

The findings also identified that while key structural factors, including the number and thickness of the remaining tooth walls, were consistently prioritised, other clinically relevant factors such as periodontal status, occlusion, tooth position, ferrule, and parafunctional habits received less consideration. These observations highlight opportunities to further strengthen comprehensive treatment planning in accordance with current evidence-based recommendations.

Overall, this study provides valuable insight into restorative decision-making using standardized clinical scenarios and supports the development of evidence-based educational strategies and future clinical research to optimise the management of root canal-treated molars.

## Data Availability

The original contributions presented in the study are included in the article/Supplementary Material, further inquiries can be directed to the corresponding author.
