# Correction: Decision-making of Jordanian dentists when restoring root canal-treated molars: a cross-sectional vignette survey

**DOI:** 10.3389/fdmed.2026.1966119

**Published:** 2026-09-17

**Authors:** Sanaa Aljamani, Rasha A. Alamoush, Aseel M. Sharaireh, Mohammad AL-Rabab’ah, Shanil Rajesh Patel, Emad Moawad, Fadi Jarad

**Affiliations:** 1 Department of Restorative Dentistry, School of Dentistry, The University of Jordan, Amman, Jordan; 2 Department of Fixed and Removable Prosthodontics, School of Dentistry, The University of Jordan, Amman, Jordan; 3 Department of Endodontics, School of Dentistry, University of Liverpool, Liverpool, United Kingdom

**Keywords:** clinical decision-making, endodontics, indirect restoration, molars, restorative dentistry, root canal treatment

Affiliation “Clinical Fellow- School of Dentistry, University of Liverpool, Amman, Jordan” was erroneously included in the paper. This affiliation has now been removed for author Sanaa Aljamani.

There was a mistake in Table 1 as published. The clinical images within Table 1 were not arranged in the intended order and were not high resolution. The corrected Table 1, with the clinical images with high resolution and arranged consistently for each scenario, appears below.

There was a mistake in the caption of Table 1 as published. The caption did not describe the intended order of the clinical images. The corrected caption of Table 1 appears below.

“TABLE 1 Represents the characteristics of each scenario: intra-oral scanner images captured after the removal of existing restoration and caries, but before the completion of RCT. For each case scenario: (a) buccal; (b) occlusal; (c) palatal/lingual; (d) bite view; and (e) periapical radiograph following endodontic completion. The coronal cavities remained unchanged in size for all cases during root canal treatment of each case (25).”

The original version of this article has been updated.

**Table:** 

	Summary of the clinical scenario	Factors involved
Scenario 1 (UR7)	Medically fit 57-year-old male with a compromised UR7 with chronic/asymptomatic apical periodontitis. The tooth was non-mobile, with no obvious cracks or localised pocketing. The patient had a class 2 division 1 occlusion and good oral hygiene, was a bruxist and did not wear a mouthguard. 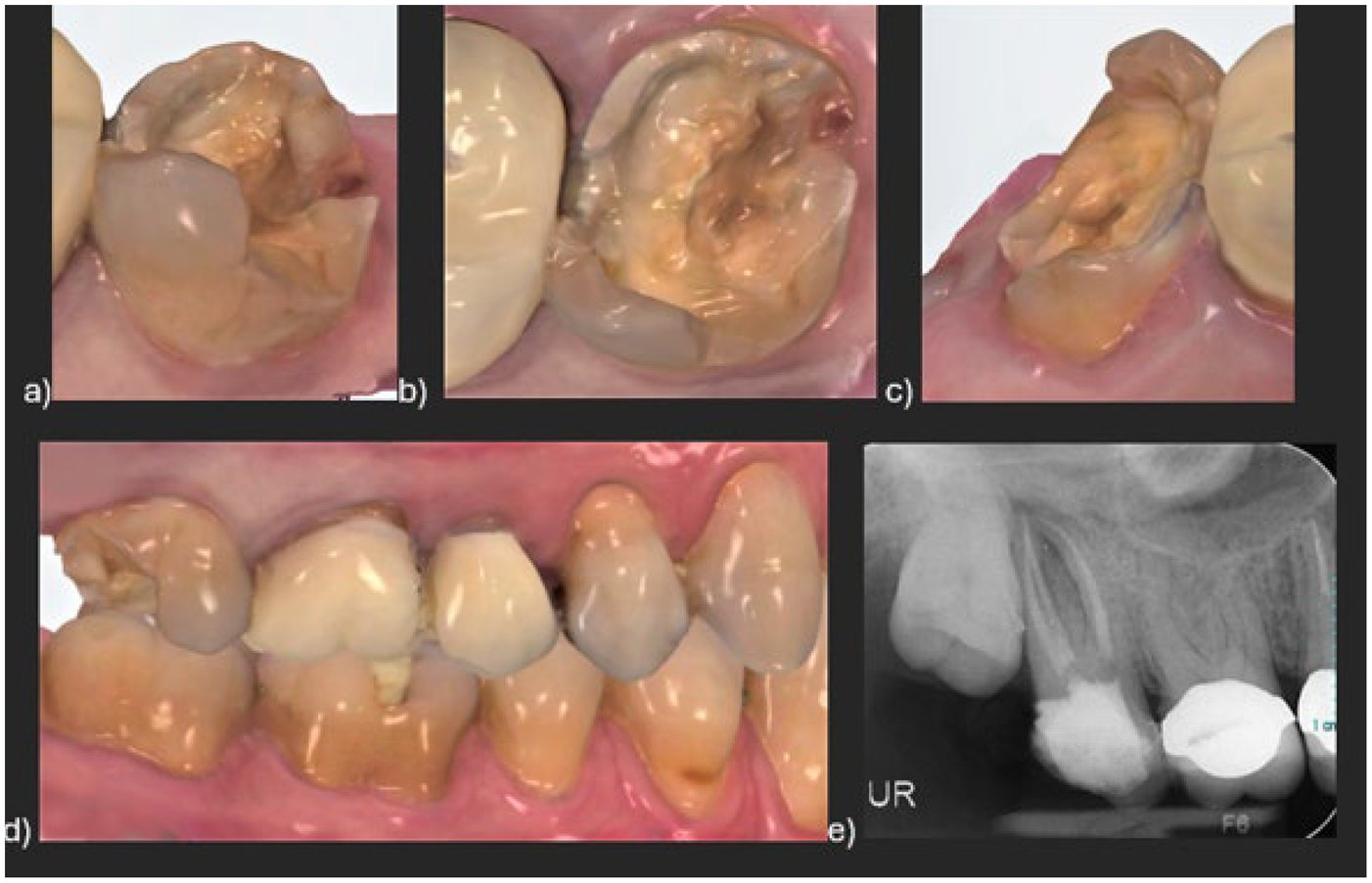	Remaining number of walls Thickness of remaining walls Parafunction Loss of proximal contacts Tooth position
Scenario 2 (LL7)	48-year-old female with well-controlled asthma presenting with chronic/asymptomatic apical periodontitis LL7. There were no clinical signs of mobility, localised pocketing or cracks, and the patient was periodontally stable. She had a class 2 division 1 occlusion and no parafunctional habits. 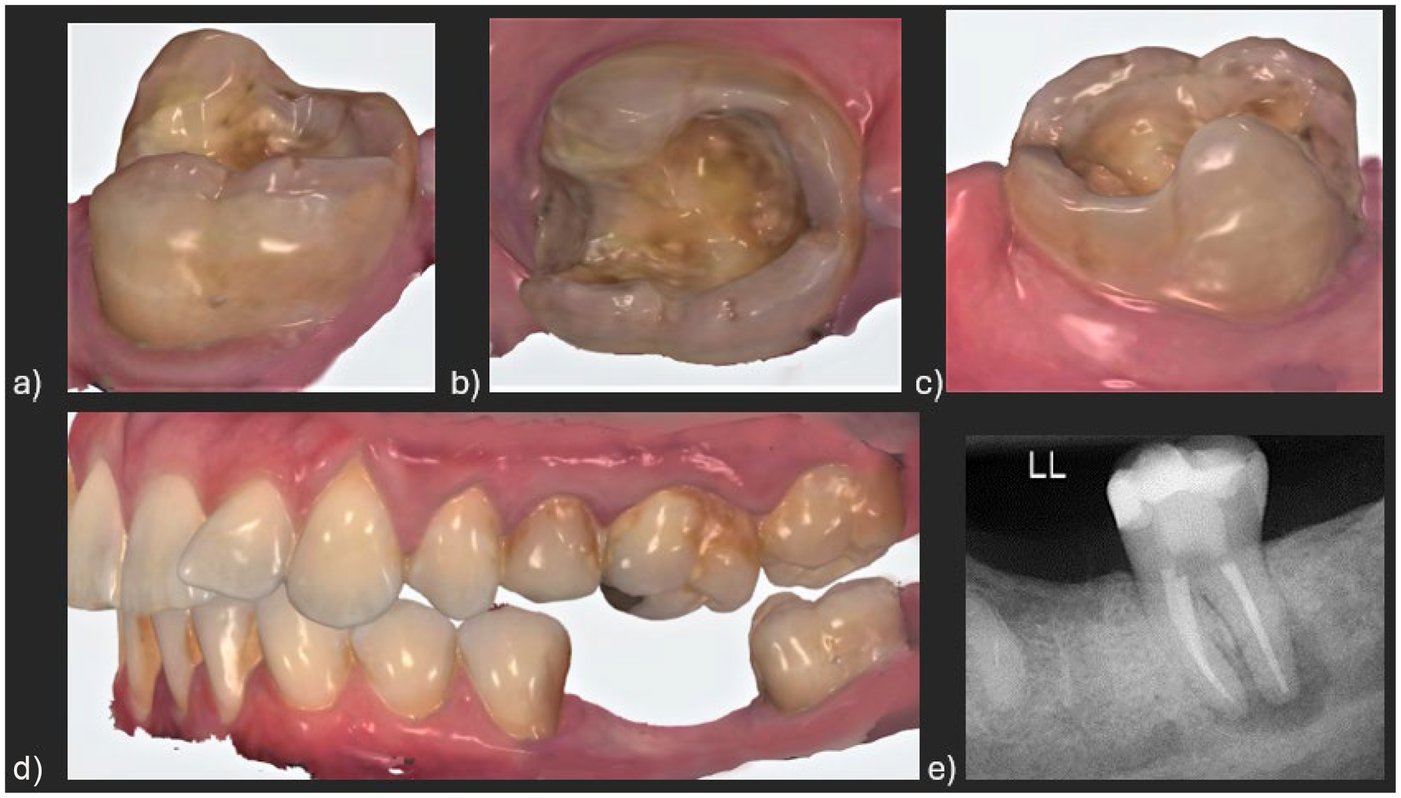	Remaining number of walls Thickness of remaining walls Loss of proximal contacts Tooth position
Scenario 3 (LL6)	57-year-old female suffering with hay fever, asthma and epilepsy had chronic/asymptomatic apical periodontitis LL6. Clinically there was no mobility or localised pocketing, but a crack was present running down the mesial margin however did not extend to the pulp chamber floor. The patient had localised plaque-induced periodontitis with BPE scores 311/120, a class 1 occlusion and did not suffer from bruxism 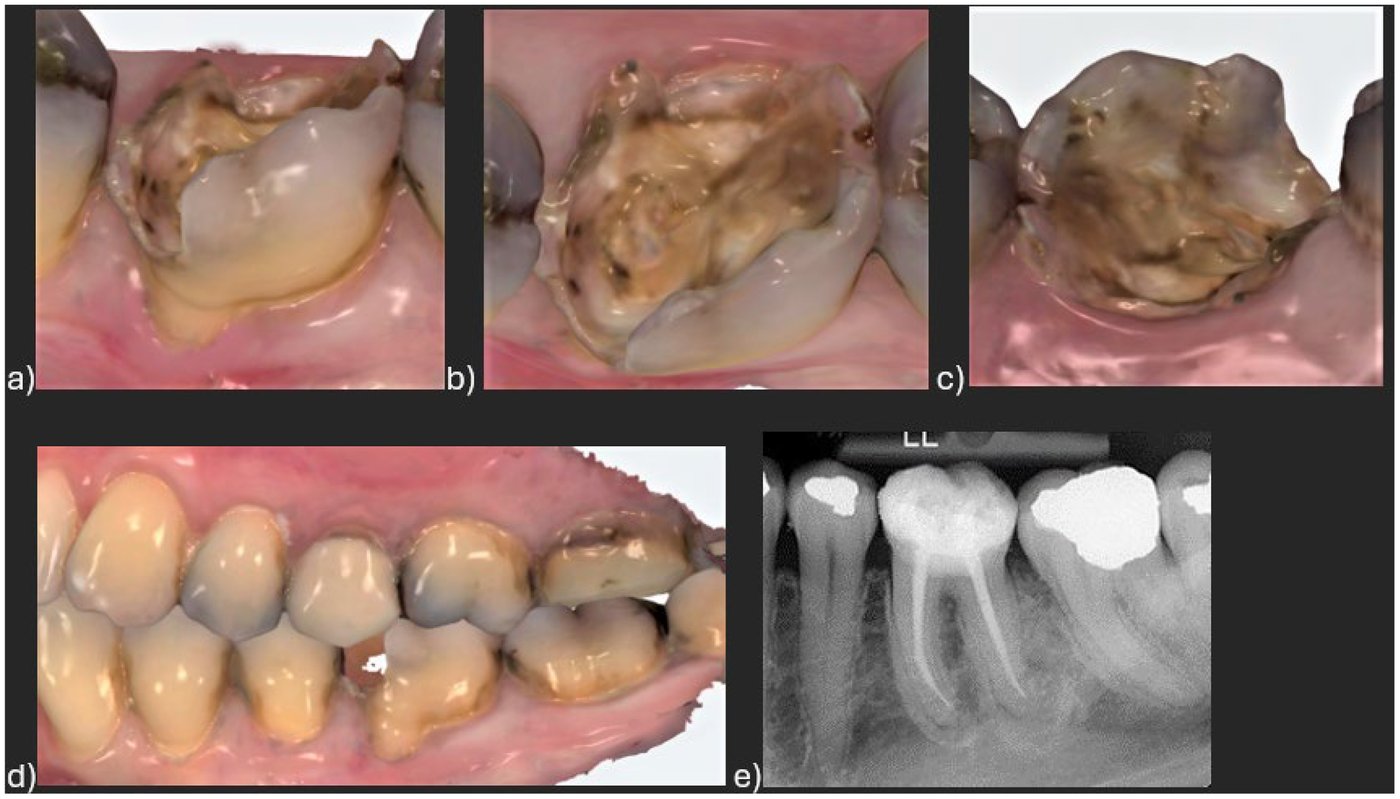	Remaining number of walls Thickness of remaining walls Cracks Periodontal health

